# Genetic Characterization and Multidisciplinary Management of Complete Androgen Insensitivity Syndrome: Unveiling a Novel AR Mutation

**DOI:** 10.1002/ccr3.72681

**Published:** 2026-05-13

**Authors:** Maria Francesca Astorino, Serena Scalise, Chiara Di Bella, Maria Angela La Rosa, Basilia Piraino, Marco Calabrò, Mattia Gentile, Rosaria Maddalena Ruggeri, Emanuela Esposito, Silvana Briuglia, Salvatore Cannavò

**Affiliations:** ^1^ University of Messina Department of Biomedical and Dental Sciences and Morpho–Functional Imaging Messina Italy; ^2^ Department of Human Pathology DETEV University of Messina Messina Italy; ^3^ Unit of Endocrinology University Hospital of Messina Messina Italy; ^4^ Unit of Genetics and Farmacogenetics University Hospital of Messina Messina Italy; ^5^ Medical Genetics Unit Bari Italy

**Keywords:** cytogenetics, endrocrinology, genetic counseling, healthcare management, metabolic disorders, molecular biological analysis

## Abstract

A novel AR frameshift mutation (c.2023_2035del) was identified in a 17‐year‐old phenotypic female with Complete Androgen Insensitivity Syndrome (CAIS). This report emphasizes the necessity of molecular characterization and multidisciplinary management to address diagnosis, surgical timing, and psychological well‐being in disorder of sex development (DSD) patients.

## 1 Introduction

Androgen Insensitivity Syndrome (AIS) is a rare X‐linked recessive disorder of sex development (DSD) [1, 2]. Population‐based estimates suggest an overall AIS prevalence of approximately 4.1 per 100,000 individuals with a 46,Xy karyotype, whereas the incidence of complete AIS (CAIS) confirmed by molecular diagnosis has been reported between 1 in 20,400 and 1 in 99,100 genetic males [2]. Also, AIS is typically characterized by evidence of feminization (i.e., undermasculinization) of external genitalia at birth, abnormal secondary sexual development in puberty, and infertility caused by the karyotype [1, 2]. It is also known as a disorder of sex development (DSD) presenting with a spectrum of defects in androgen action results from hormone resistance caused by in the X‐linked androgen receptor (*AR*) gene [1]. Depending on the severity of androgen resistance, AIS can be subdivided into Complete Androgen Insensitivity Syndrome (CAIS), Partial Androgen Insensitivity Syndrome (PAIS) or Mild Androgen Insensitivity Syndrome (MAIS) [1, 2] (Table 1).

**TABLE 1:** Summary of the clinical characteristics of the three broad phenotypes of AIS.

	CAIS	PAIS	MAIS
Genitalia	Female	Predominantly female/male	Male
Pubic hair	Absent or scant	Normal or reduced	Normal
Clitoral enlargement	(−)	(+)	n/a
Testis	Abdominal/inguinal, labial	Descended or undescended testes	Descended testes
Mammary development	Normal female	Gynecomastia in puberty	Gynecomastia in puberty

CAIS has a prevalence ranging from 1 in 20,400 to 1 in 99,100 [3]. A patient affected by CAIS has 46,XY chromosome and complete inability to respond to androgens [1]. Its diagnosis depends on three situations: during fetal life, is identified 46,XY karyotype with the presence of female external genitalia; in childhood the young girl may possibly have an inguinal hernia, or more frequently primary amenorrhea during puberty [3]. Patients with CAIS develop breasts due to the lack of androgen action, despite estradiol levels being within the normal male range, rather than increased estrogen production. Menstrual cycles are absent because testicular production of anti‐müllerian hormone (AMH) prevents the development of the uterus, cervix, and upper vagina. Most patients have a shortened, blind‐ending vagina (2.5–8 cm in CAIS; 1.5–4 cm in PAIS), and pubic and axillary hair is sparse or absent [3].

The PAIS clinical phenotype depends on the degree of *AR* residual function and ranges from proximal hypospadias to micropenis [3]. Hypospadias is one of the most common congenital urogenital malformations in males, with reported prevalences in the order of tens of cases per 10,000 live male births, varying by population and ascertainment method [3]. PAIS is indicated by gynecomastia during puberty in patients with atypical genitalia [3].

MAIS is associated with *AR* mutations, but patients are without external genitalia abnormalities. The investigation of male infertility or pubertal gynecomastia helps to diagnose MAIS. Pathogenic expansion of the CAG trinucleotide repeat in exon 1 of the *AR* gene underlies spinal and bulbar muscular atrophy (SBMA, Kennedy disease), an X‐linked neuromuscular disorder that combines lower motor neuron degeneration with clinical features of androgen insensitivity. SBMA should be distinguished from mild AIS (MAIS), in which external genitalia are typically normal and androgen resistance primarily manifests as infertility or gynecomastia [3]. Normal male external genitalia, testosterone resistance develops with disease progression in MAIS [3].

## 2 Case History/Examination

Herein we describe the clinical, endocrinological, and molecular features of a 17‐year‐old individual with a 46,XY karyotype and a female external phenotype consistent with CAIS. The patient, female from the phenotypic point of view, referred to the Endocrine Unit of “G. Martino” Hospital University for evaluation of primary amenorrhea. At first evaluation, a detailed history of the patient was taken along with a physical examination. Her family medical history is unremarkable. On physical examination, she was of normal weight (height: 155 cm, weight: 61 kg; BMI: 25.39 kg/m^2^). Breast development was consistent with Tanner stage B3; axillary hair was absent, and pubic hair was at Tanner stage P2. External genitalia appeared normal (female phenotype). A pelvic ultrasound performed at an external center, presented by the patient at referral, was initially interpreted as showing “ovaries in place” but failed to visualize the uterus. In retrospect, and considering subsequent imaging and genetic findings, these structures most likely corresponded to intra‐abdominal testes rather than true ovaries.

Biochemical evaluation of the hypothalamic–pituitary–gonadal axis showed elevated LH (25.80 mIU/mL) with relatively normal FSH (2.46 mIU/mL), low‐normal estradiol (28 pg/mL), and markedly increased total testosterone (633 ng/dL), together with elevated AMH, delta‐4‐androstenedione, and DHEAS, and normal SHBG (Table 2). DHEAS was 497 μg/dL(Table 2).

**TABLE 2:** Biochemical data in the female case.

	Patients values	Normal values
FSH	2.46 mIU/mL	(male 1.5–12.4 female follicular phase 3.5–12.5)
LH	25.80 mlU/mL	(male 1.7–8.6 female follicular phase 2.4–12.6)
17 beta‐estradiol (E2)	28 pg/mL	(male 11.3–43.2 female follicular phase 12.4–233)
Total testosterone	633 ng/dL	(male 163–836 female 2.9–48.1)
Delta‐4 ANDROSTENEDIONE	3.70 ng/mL	(male 0.5–4.8 female follicular phase 0.9–3.0)
Dehydroepiandrosterone sulfate (DHEAS)	497 μg/dL	(male 16.1–492 female 9.4–407)
Anti‐Müllerian hormone (AMH)	18.10 ng/mL	(0.0–2.4)
Sex hormone binding globulin (SHBG)	48.7 nmol/L	(male 9–55 female 20–85)

Pelvic ultrasound, repeated in our outpatient clinic, revealed two solid, oval‐shaped structures with homogeneous, hypoechoic echotexture and cranially located anechoic cystic components. The uterus was not identified. Abdominal MRI confirmed two oval formations with regular margins and heterogeneous signal intensity, measuring 43 × 30 × 21 mm on the right and 40 × 26 × 13 mm on the left, each associated with cranial cystic structures (20 mm and 18 mm, respectively). These findings were highly suggestive of intra‐abdominal testes and prompted molecular testing.

## 3 Informed Consent

Written informed consent was obtained from the patient for the publication of this case report and any accompanying images.

## 4 Differential Diagnosis

The primary differential diagnosis for a phenotypic female presenting with primary amenorrhea and absent Müllerian structures includes Mayer–Rokitansky–Küster–Hauser (MRKH) syndrome and Androgen Insensitivity Syndrome (CAIS). Distinction relies on karyotype and hormonal profile: MRKH patients exhibit a 46,XX karyotype with normal ovarian function, whereas CAIS patients present with a 46,XY karyotype and male‐range testosterone levels.

Other conditions to consider include 17β‐hydroxysteroid dehydrogenase deficiency and 5α‐reductase deficiency. However, the latter typically presents with virilization at puberty, which was absent in our patient. Swyer syndrome (46,XY gonadal dysgenesis) was excluded due to the presence of functional testicular tissue and elevated testosterone levels. In this case, the combination of a 46,XY karyotype, female phenotype with absent axillary hair, and a pathogenic AR mutation confirmed the diagnosis of CAIS.

## 5 Conclusion and Results

The following genetic analyzes have been carried out:

- Analysis of the karyotype from peripheral blood: the karyotype has been obtained by T–lymphocytes extracted from peripheral blood. After obtaining metaphases from cell culture, the chromosomes of metaphases were stained by the G‐banding method with a resolution of 400–550 bands.
- Molecular study focused on the determination of the presence/absence of the SRY gene [located on the short arm of the chromosome (Yp11.3)] and of specific STS (Sequences Tag Sites) located in the regions AZFa, AZFb, and AZFc of the long arm of Y chromosome and studied for the screening of the microdeletions of Y chromosome. Analysis is performed due to DNA, obtained by T–lymphocytes extracted by peripheral blood. The molecular analysis included:
  ○ DNA was extracted from peripheral blood (200 μL) using QIAwave DNA Blood & Tissue Kit (Qiagen);
  ○ DNA was quantified using a Qubit 4 Fluorometer (Thermo Fisher Scientific, Waltham, Massachusetts, USA) according to the manufacturer's protocol;
  ○ DNA sample was amplified using Devyser AZF v2 and extension CE‐IVD;
  ○ The amplified was directly sequenced using Devyser Dye–Set DEV–5: 560 Sizer Orange and ABI 3500 Genetic Analyzer (Applied Biosystem), a fully automated fluorescence‐based capillary electrophoresis platform.
- Analysis of clinical exome sequencing from peripheral blood was performed at “UOC Laboratorio Genetica medica—ASL Bari (Puglia)”. DNA has been obtained by T–lymphocytes extracted by peripheral blood. The analysis is performed by sequencing all coding regions of genes known to be associated with AIS.

### 5.1 Molecular Results

The result coming from molecular studies has confirmed the suggestion of AIS.

**FIGURE 1:**
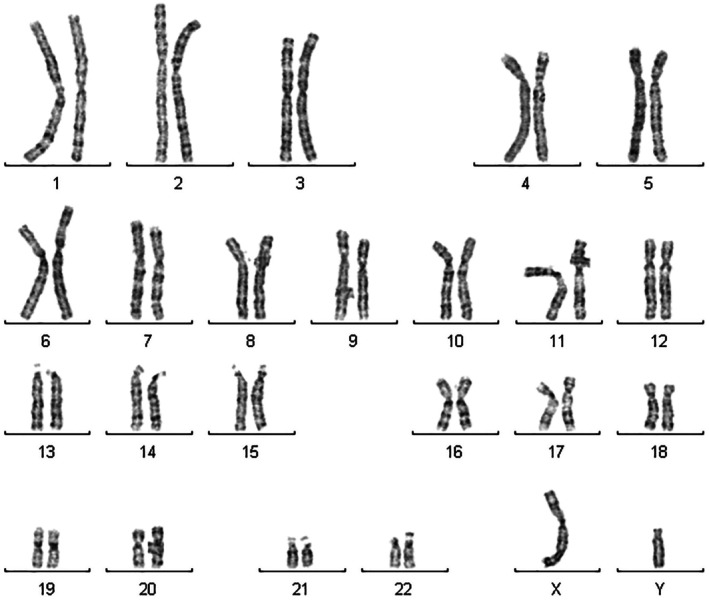
The karyotype of the patient showed a 46, XY chromosomal pattern.

The karyotype investigation highlighted a regular male karyotype (Figure 1).

**FIGURE 2:**
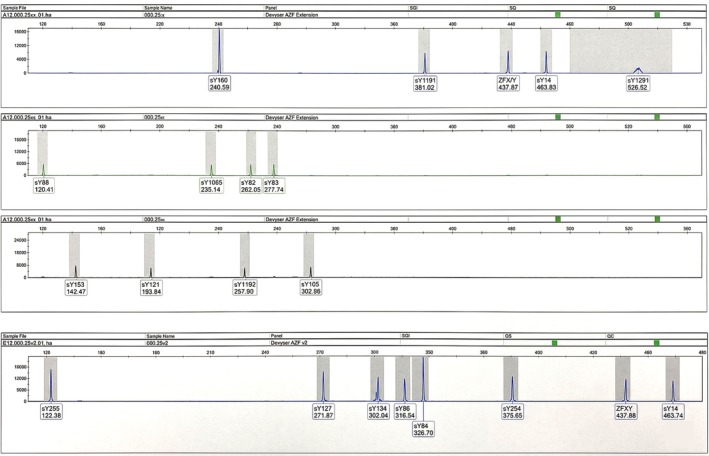
The analyzes of STS markers in the AZFa, AZFb, and AZFc regions of the Y‐chromosome showed a normal male sample.

The molecular analysis has confirmed the presence of the Y chromosome and highlighted the presence of the SRY gene (Figure 2), consistent with the 46,XY karyotype, and showed an intact AZF region on the long arm of the Y chromosome (Figure 2). In line with current DSD terminology, these findings support the diagnosis of a 46,XY disorder of sex development consistent with CAIS.

Sequence analysis identified the following hemizygous variant in the *AR* gene (NM_000044.6): c.2023_2035delCTGAATGTCCTGG, a frameshift deletion in exon 4 that results in an altered reading frame and premature termination of the protein (p.Leu675LysfsTer7). The present variant is not reported in the ClinVar database and also this is the first description in the literature. Given that this frameshift variant is expected to result in nonsense‐mediated decay of the AR transcript in a gene with a well‐established loss‐of‐function disease mechanism, and considering its absence from population databases and deleterious in silico predictions, we classified it as a pathogenic variant according to ACMG/AMP criteria. In particular, the variant meets PVS1 (null variant in a gene where loss of function is a known disease mechanism), PM2_supporting (absence from population allele frequency databases), and PP4 (phenotype highly specific for disease with a single genetic etiology). Variant interpretation followed the ACMG/AMP standards and guidelines for the interpretation of sequence variants [4]. Pathogenic variants in *AR* are generally associated with AIS (AIS, OMIM #300068) with X‐linked recessive inheritance (XLR). To establish whether the variant found in the patient is de novo or transmitted by maternal chromosome, blood mother's patient was also taken the familial variant of the *AR* gene searched for. Sequence analysis identified the variant sought in heterozygosity in the *AR* gene (NM_000044.6): c.2023_2035del CTGAATGTCCTGG (p.Leu675LysfsTer7), already identified in the daughter.

### 5.2 Outcome and Follow‐Up

Genetic counseling plays a central role in informing the patient and family about inheritance patterns, reproductive options, and long‐term follow‐up.

The identification of a genetic disorder has implications not only for the individual patient but also for the wider family, and counseling before and after testing is essential to evaluate reproductive risks and to provide emotional and psychosocial support. In particular, counseling interventions can help mitigate feelings of maternal guilt in cases where the mother is a carrier of an AIS‐related variant and can facilitate adaptation to the diagnosis.

During posttest genetic counseling, we explained the X‐linked recessive inheritance of CAIS and the identification of the *AR* variant in the proband's mother, who is a heterozygous carrier. We recommended cascade testing and dedicated counseling for at‐risk female relatives on the maternal side (e.g., sisters and maternal aunts), with the aim of clarifying their carrier status, supporting informed reproductive decision‐making, and offering ongoing psychosocial support. These recommendations were integrated into the family's long‐term follow‐up plan.

The patient was raised as female and currently self‐identifies as female. As part of the multidisciplinary DSD team, a psychologist with expertise in gender development provides ongoing support, addressing issues related to gender identity, body image, and disclosure at a pace acceptable to the patient. These assessments have informed shared decision‐making regarding the timing of gonadectomy, hormonal management, and future reproductive counseling.

In our adolescent patient, gonadectomy has been deferred to allow spontaneous pubertal progression and to ensure that she can actively participate in decisions regarding fertility, body image, and hormone replacement. She is currently followed in a multidisciplinary DSD clinic with annual clinical review and periodic imaging surveillance (pelvic ultrasound and/or MRI) of the intra‐abdominal gonads. This protocol may be intensified if new symptoms, imaging abnormalities, or tumor markers suggest an increased risk of gonadal pathology.

## 6 Discussion

AR is a member of the steroid hormone–thyroid hormone–retinoic acid receptor family and plays a crucial role in male development. The *AR* gene, located on the long arm of the X chromosome (Xq11–12), spans approximately 90 kilobases (kb) and is composed of eight exons (A–H or 1–8) separated by seven introns [5].

The *AR* gene encodes a modular protein with distinct functional domains. The DNA‐binding domain (DBD) enables interaction with specific androgen‐responsive elements (AREs) in target genes, while the ligand‐binding domain (LBD) ensures high‐affinity binding to androgens [6]. Additionally, the open reading frame (ORF) encodes a large N‐terminal domain, which is essential for maximal transcriptional activation [7].

This gene encodes the AR protein, which is composed of 919 amino acids and has a molecular weight of approximately 110 kilodaltons (kDa). The protein features four distinct functional domains: N‐terminal Transactivation Domain (TAD) plays a crucial role in activating gene transcription; DNA‐Binding Domain (DBD) facilitates direct interaction with the DNA sequences of target genes; Hinge Region provides structural flexibility and connects the DBD to the ligand‐binding domain; Ligand‐Binding Domain (LBD) serves as the binding site for steroid hormones, enabling receptor activation and subsequent transcriptional regulation [5, 6].

Functionally, AR acts as an intracellular transcriptional activator, regulating the expression of androgen‐responsive genes [6]. These structural features enable AR to mediate critical physiological functions by modulating gene expression in response to hormonal signals [5].

The *AR* gene is essential for androgenic signaling, orchestrating gene regulation in target tissues [3, 7]. Mutations in the *AR* gene are directly associated with abnormalities in male sexual development, leading to a spectrum of AIS. Over 1000 *AR* coding sequence variants have been identified, each contributing to varying degrees of cellular insensitivity to androgens. These mutations primarily affect the *AR* open‐reading frame or induce single amino acid substitutions, predominantly within the DNA‐ or ligand‐binding domains, ultimately disrupting receptor function [3, 7]. Notably, approximately 30% of AR mutations in AIS are de novo, underscoring the importance of comprehensive AR sequencing in all 46,XY disorders of sex development (DSD) newborns, regardless of family history.

The distribution of mutations varies across the *AR* gene. Exon 1, which encodes the NTD, harbors the highest mutation frequency and is commonly affected in CAIS. Conversely, mutations in exons 5 and 6, which encode the LBD, are more frequently associated with PAIS. Disruptions in the reading frame typically result in CAIS, where 46,XY individuals develop female external genitalia despite the presence of undescended testes. In contrast, missense mutations affecting the DNA‐ or ligand‐binding domains give rise to a continuum of androgen–resistant phenotypes, ranging from mild undervirilization to infertility [3, 5, 7, 8]. Notably, a deletion of exon 4 has been reported in a phenotypic male with azoospermia, highlighting the variable expressivity of *AR* mutations.

Among the most prevalent *AR* allelic variants in AIS are nonsynonymous point mutations, while insertions and deletions leading to frameshift mutations and premature stop codons are predominantly found in CAIS. Variants affecting mRNA splicing are reported across both CAIS and PAIS, with rare cases of synonymous mutations altering splicing sites. Large structural rearrangements, including deletions of exons or the entire *AR* gene, have been documented but are exceedingly rare. Genetic studies on families with suspected AR dysfunction, based on endocrine profiling and inheritance patterns, reveal that mutations most frequently localize to the DNA‐binding domain, primarily manifesting as single amino acid substitutions. Although these mutant receptors often retain androgen‐binding capacity, they exhibit defective transcriptional activation, as demonstrated in vitro assays using androgen‐responsive reporter genes [3, 7].

Despite extensive genotype–phenotype correlations, AIS exhibits significant clinical variability, with some *AR* variants associated with multiple phenotypic outcomes. This variability remains incompletely understood but is hypothesized to involve AR co‐regulators (activators and repressors), differential 5α‐reductase Type 2 activity influencing dihydrotestosterone (DHT) availability, and the presence of germline *AR* variants at a postzygotic stage, leading to somatic mosaicism. These factors underscore the complex molecular mechanisms governing *AR* function and the phenotypic heterogeneity observed in AIS [3]. Somatic mosaicism of *AR* variants has been reported as a potential contributor to the broad phenotypic spectrum of AIS. In our case, the uniform CAIS phenotype and analysis of leukocyte‐derived DNA did not suggest mosaicism, although low‐level somatic mosaicism in other tissues cannot be completely excluded [9].

AIS results from impaired androgen receptor (AR)–mediated signaling. Depending on the underlying variant, AR dysfunction may involve reduced or absent ligand binding, defective nuclear translocation, impaired interaction with co‐regulators, or loss of transcriptional activity, leading to a broad spectrum of androgen resistance phenotypes [10, 11].

In 46,XY fetuses, testicular development is initiated by the *Sex–Determining Region Y* (SRY) gene. From the second month of gestation, the fetal testes secrete two critical hormones: anti‐Müllerian hormone (AMH) and testosterone. AMH, secreted by Sertoli cells, inhibits the development of Müllerian ducts, thereby preventing the formation of female internal reproductive structures such as the uterus [5, 11]. Concurrently, Leydig cells produce testosterone, the principal androgenic hormone in humans. Testosterone enters target cells via passive diffusion, where it partly converts to 5α‐dihydrotestosterone (DHT) through the action of 5α‐reductase. DHT, with a higher binding affinity for the AR than testosterone, is critical for androgenic signaling [5, 11, 12].

During embryogenesis, testosterone acts on Wolffian ducts to promote differentiation into internal male reproductive structures (epididymis, vas deferens, seminal vesicles), while DHT is essential for the development of external male genitalia [5, 11]. Both testosterone and DHT bind to the AR, inducing a conformational change that enables AR dimerization and subsequent binding to androgen response elements (AREs) within the genome. This receptor‐ligand complex initiates transcription of androgen–responsive genes. In the absence or dysfunction of the AR, this pathway is disrupted, rendering androgens biologically inactive and resulting in androgen insensitivity [5, 11].

In AIS the endocrine profile reflects androgen resistance, with elevated or normal basal testosterone levels and increased serum LH levels [13]. High serum AMH and testosterone levels in newborns suggest AIS and exclude complete gonadal dysgenesis [14]. In postpubertal patients, estradiol levels are normal or slightly elevated compared to male reference ranges [13]. This hormonal pattern is observed during mini‐puberty or after puberty. During childhood, when the gonadotropin axis is inactive, hCG stimulation is required to assess Leydig cell testosterone secretion.

The elevated basal testosterone and LH levels in AIS indicate disrupted androgen‐mediated negative feedback on the anterior pituitary [13]. FSH levels typically remain normal due to regulation by gonadal inhibin [15]. Despite differences in AR function between CAIS and PAIS phenotypes, no significant differences are observed in serum LH, FSH, estradiol, or DHT levels [13]. Hormone levels in the three broad phenotypes of AIS are reported below (Table 3) [10, 16, 17].

**TABLE 3:** Hormonal profiles in CAIS, PAIS, MAIS individuals.

	CAIS	PAIS	MAIS
FSH	N (within adult male reference range) or high‐normal, usually not markedly elevated	Mostly N	Usually N
LH	↑ (increased relative to adult male range), typically above male reference range because of absent androgen feedback	↑ (pattern similar to CAIS)	Often N, but can be ↑ in some patients
17 beta‐estradiol (E2)	N or slightly ↑ relative to males, often ≈20–40 pg/mL	N or slightly ↑ versus males	Male‐range (low)
Total testosterone	↑, often in the mid‐ to high‐male range or above	N–↑, often mid‐ to high‐male range	Mostly N; sometimes ↑ in the setting of infertility or gynecomastia
Delta‐4 ANDROSTENEDIONE	Usually N within male range	Data limited; typically within male range	Within male range
Dehydroepiandrosterone sulfate (DHEAS)	Often ↓ (decreaded relative to adult male range) or low‐normal	Data limited; generally within normal range	Within male range
Anti‐Müllerian hormone (AMH)	N or ↑ (functional Sertoli cells; high in infancy)	N or ↑	N or mildly ↑; generally not diagnostic
Sex hormone binding globulin (SHBG)	N–↑, frequently in the female reference range (≈50 nmol/L)	Likely N–↑; specific data limited, often similar to CAIS	Typically N; not a characteristic abnormality

Our patient's endocrine profile, characterized by elevated LH, relatively normal FSH, male‐range testosterone, and low‐normal estradiol levels, is consistent with androgen resistance. Elevated LH reflects impaired androgen‐mediated negative feedback at the hypothalamic‐pituitary axis, whereas FSH remains within the normal range due to preserved regulation by inhibin and estradiol [18]. In CAIS, high circulating testosterone is peripherally aromatized to estradiol, which supports spontaneous breast development despite the absence of uterine structures and menstrual bleeding [18]; this mechanism likely underlies the Tanner B3 breast development observed in our patient.

According to literature, AIS and particularly those patients with CAIS show an increased risk of developing a long‐term consequence [19]. In addition to impaired fertility, there is an elevated risk of developing cardiovascular diseases, obesity, metabolic disorders such as diabetes, and various psychiatric conditions.

DSDs are recognized as risk conditions for the development of germ cell tumors [19], both seminomatous and nonseminomatous (yolk sac tumors, choriocarcinoma, embryonal cell carcinoma, etc.) [20]. Various pathogenetic hypotheses raise concerns regarding the presence of the Y‐chromosome encoded testis‐specific protein, which regulates the differentiation of germ cells [21, 22].

The likelihood of germ cell tumors in CAIS remains low but rises progressively after puberty [3]. Historically, most individuals with CAIS underwent prophylactic gonadectomy during childhood or early adolescence, resulting in scarce long‐term data on intra‐abdominal gonads in adult patients [23]. Currently, from different approaches, gonadectomy is a solution until late adolescence—once spontaneous pubertal maturation is complete—and up to 15% of individuals with CAIS retain their gonads [24]. Those who preserve their gonads during puberty often achieve enhanced, even normative, bone mineral density and spontaneous breast development owing to peripheral aromatization of androgens into estrogens [25]. Moreover, the choice to proceed with gonadectomy can be made by the patient rather than by their parents early in life [26]. Surveillance of retained gonads is performed via ultrasound for inguinal or labioscrotal testes and by magnetic resonance imaging for intra‐abdominal or extra‐abdominal gonads [27]. However, these imaging modalities lack sufficient specificity to detect in situ germ cell tumors [27]. Classical serum markers—beta‐human chorionic gonadotropin and alpha‐fetoprotein—are typically elevated in nonseminomatous tumors but not in seminomas, which are the predominant germ cell tumor subtype in CAIS, rendering them unreliable for monitoring [28]. Emerging screening strategies, including assays for specific microRNAs and single‐nucleotide polymorphisms, are under investigation.

The body resistance to the effects of androgens may cause negative effects also in bone development and maintenance [29]. This form of resistance can lead to a reduction in bone mineral density (BMD), which is different between women with intact gonads (CAIS), with a normal BMD, and those who have had their gonads removed; often, they experience decreased BMD, particularly in the lumbar spine and femoral neck [29]. Androgens, such as testosterone, are pivotal regulators of skeletal development, growth, and homeostasis through their modulation of bone remodeling—the coordinated processes of resorption and formation [29, 30]. Sex steroids not only act on the maturing skeleton to preserve structural integrity but also drive the emergence of sex‐specific differences in bone architecture beginning at puberty. Their actions are chiefly mediated by activation of peripheral hormone receptors—namely AR and estrogen receptor‐α (ER‐α)—which are expressed in osteoblasts, osteoclasts, osteocytes, and growth–plate chondrocytes within an age‐ and sex‐dependent endocrine milieu [29]. Additionally, distinct effects of estrogens and androgens on skeletal muscle function and sensitivity further shape bone health and contribute to sexual dimorphism.

A small or underdeveloped vagina is a common feature in CAIS [31]. In these patients, the absence of the uterus and fallopian tubes is primarily due to anti‐Müllerian hormone (AMH) produced by Sertoli cells, which induces regression of Müllerian ducts during fetal life. The lower portion of the vagina derives from the urogenital sinus and is typically present but hypoplastic, resulting in a shortened blind‐ending vaginal canal that may impair penetrative intercourse [31]. The ensuing vaginal hypoplasia often impairs penetrative intercourse due to an insufficiently sized or shortened vaginal canal. Initial management typically involves nonsurgical dilator therapy to promote canal elongation and elasticity. In refractory cases, surgical creation or augmentation of the vaginal tract (vaginoplasty) may be undertaken; however, these procedures are technically demanding and generally require long‐term postoperative dilation to maintain patency [32].

In CAIS, the risk of gonadal germ cell tumors is considered very low in childhood but increases with age. A systematic review of 15 studies reported premalignant lesions in approximately 6% and malignant lesions in about 1%–2% of CAIS patients undergoing gonadectomy or biopsy, with most lesions identified after puberty [33]. Other series and modeling studies have suggested that the cumulative risk of gonadal malignancy may reach 3%–14% in adulthood, with considerable heterogeneity across cohorts [18, 33, 34]. These data support a shared decision‐making approach that balances the relatively low malignancy risk against the benefits of retaining gonads for spontaneous pubertal development and bone health.

In recent years, management of individuals with AIS has predominantly involved hormone replacement therapy (HRT) alongside psychosocial support [25, 35]. In those who retain their gonads, CAIS do not require immediate endocrine intervention, since spontaneous pubertal development typically occurs in line with peers due to sufficient estradiol production via peripheral aromatization of testicular androgens [25]. Nevertheless, when HRT is indicated, it is usually initiated around 11–12 years of age using estrogen formulations (or testosterone in cases of partial AIS in individuals assigned male at birth) to induce normal secondary sexual characteristics, support the pubertal growth spurt, and promote appropriate skeletal maturation [36]. The goal of therapy is to ensure the normal development of secondary sexual characteristics, enable the pubertal growth spurt, and promote skeletal maturation [36]. There are no AIS‐specific induction protocols; instead, clinicians apply general guidelines for pubertal induction in hypogonadal patients of any etiology, beginning with low hormone doses and gradually escalating based on circulating hormone measurements and clinical markers of progression [25, 35]. More recently, the concept of using testosterone replacement in CAIS has been investigated, with the rationale that reinstating pregonadectomy androgen levels—albeit recognizing peripheral aromatization—is a potentially more physiological approach to HRT in this population [25, 35, 36].

Although CAIS is a rare X‐linked recessive condition [24], it must be considered in the differential diagnosis of primary amenorrhea, particularly in patients presenting with a female phenotype, 46,XY karyotype, and absent Müllerian structures. The diagnostic approach should include a thorough clinical evaluation, hormonal profiling, imaging studies, and cytogenetic and molecular analysis to confirm the diagnosis and identify mutations in the androgen receptor gene. Early diagnosis is crucial, not only for appropriate clinical management but also for psychological support and long‐term care planning. While gonadectomy was traditionally recommended in early adolescence, current guidelines suggest delaying the procedure until after puberty to allow for spontaneous feminization under endogenous estrogen production [37]. However, the timing of gonadectomy must be carefully individualized, as retained intra‐abdominal testes carry a low but nonnegligible risk of malignant transformation that appears to increase with age, with small series suggesting a cumulative risk in the low single‐digit to low double‐digit percentage range, and substantial heterogeneity across cohorts [33, 38, 39]. Long‐term follow‐up should include hormone replacement therapy, bone health monitoring, and psychological support to optimize quality of life. Given the rarity and complexity of the condition, CAIS highlights the importance of early recognition and the value of integrated, patient‐centered care.

Management of CAIS requires a coordinated, multidisciplinary team—including endocrinologists, gynecologists, geneticists, and mental health professionals to address both the medical and psychosocial implications of the diagnosis.

## Author Contributions

**Maria Francesca Astorino:** conceptualization, formal analysis, investigation, visualization, writing – original draft, writing – review and editing. **Serena Scalise:** conceptualization, investigation, writing – original draft. **Chiara Di Bella:** formal analysis, investigation. **Maria Angela La Rosa:** formal analysis, investigation, resources. **Basilia Piraino:** resources. **Marco Calabrò:** conceptualization, resources. **Mattia Gentile:** formal analysis, investigation, methodology, resources, validation. **Rosaria Maddalena Ruggeri:** conceptualization, investigation, resources, supervision, writing – original draft. **Emanuela Esposito:** resources, supervision. **Silvana Briuglia:** conceptualization, investigation, project administration, supervision. **Salvatore Cannavò:** investigation, project administration, supervision.

## Funding

The authors have nothing to report.

## Consent

Written informed consent was obtained from the patient to publish this report in accordance with the journal's patient consent policy.

## Conflicts of Interest

The authors declare no conflicts of interest.

## Data Availability

All relevant data supporting the findings of this study are included within the article. No personally identifiable information has been reported, in accordance with ethical and privacy regulations.
