# Correction to “Genetic Characterization and Multidisciplinary Management of Complete Androgen Insensitivity Syndrome: Unveiling a Novel AR Mutation”

**DOI:** 10.1002/ccr3.73043

**Published:** 2026-06-28

A. M. Francesca , S. Serena , D. B. Chiara , et al., “Genetic Characterization and Multidisciplinary Management of Complete Androgen Insensitivity Syndrome: Unveiling a Novel AR Mutation,” Clinical Case Reports 14, no. 5 (2026): e72681, 10.1002/ccr3.72681.42137628 PMC13169139

The first and last names of the authors were inadvertently swapped and have been corrected.

The correct author names are: Maria Francesca Astorino, Serena Scalise, Chiara Di Bella, Maria Angela La Rosa, Basilia Piraino, Marco Calabrò, Mattia Gentile, Rosaria Maddalena Ruggeri, Emanuela Esposito, Silvana Briuglia, and Salvatore Cannavò.

The online version of this article has been corrected accordingly.

We apologize for this error.

